# Effectiveness of allied health clinical supervision following the implementation of an organisational framework

**DOI:** 10.1186/s12913-022-07636-9

**Published:** 2022-02-26

**Authors:** Marcus J. Gardner, Carol McKinstry, Byron Perrin

**Affiliations:** 1 La Trobe Rural Health School, La Trobe University, P.O. Box 199, Victoria 3552 Bendigo, Australia; 2 Bendigo Health, P.O. Box 126, Bendigo, Victoria 3552 Australia

## Abstract

**Background:**

Clinical supervision makes an important contribution to high quality patient care and professional wellbeing for the allied health workforce. However, there is limited research examining the longitudinal implementation of clinical supervision for allied health. The aim of this study was to determine the effectiveness of clinical supervision for allied health at a regional health service and clinicians’ perceptions of the implementation of an organisational clinical supervision framework.

**Methods:**

A cross-sectional study was conducted as a phase of an overarching participatory action research study. The Manchester Clinical Supervision Scale (MCSS-26) tool was used to measure clinical supervision effectiveness with additional open-ended questions included to explore the implementation of the clinical supervision framework. MCSS-26 findings were compared with an initial administration of the MCSS-26 5 years earlier. MCSS-26 data (total scores, summed domain and sub-scale scores) were analysed descriptively and reported as mean and standard deviation values. Differences between groups were analysed with independent-samples t-test (t) and one-way between groups ANOVA.

**Results:**

There were 125 responses to the survey (response rate 50%). The total MCSS-26 score was 78.5 (S.D. 14.5). The total MCSS-26 score was unchanged compared with the initial administration. There was a statistically significant difference in clinical supervision effectiveness between speech pathology and physiotherapy (F = 2.9, p = 0.03) and higher MCSS-26 scores for participants whose clinical supervisor was a senior clinician and those who chose their clinical supervisor. Seventy percent of participants perceived that the organisation’s clinical supervision framework was useful and provided structure and consistent expectations for clinical supervision.

**Conclusions:**

Clinical supervision was effective for allied health in this regional setting and clinical supervision effectiveness was maintained over a 5 year period. The implementation of an organisational clinical supervision framework may have a positive effect on clinical supervision for some professions.

## Background

Allied health clinicians play a vital role in the provision of high quality patient care across the continuum of care in health care systems [1, 2]. The allied health workforce are a significant proportion of the health workforce, ranking as the largest workforce behind nursing, and medicine in some settings [3]. Professions included as allied health disciplines differs across settings and countries. The professionals commonly described as allied health include physiotherapy, occupational therapy, speech pathology, podiatry, dietetics and, exercise physiology [4–6]. Additionally, in some contexts social work, psychology and allied health assistants are also defined as allied health [7]. Across the majority of allied health professions, clinical supervision is practised to enhance the professional development of clinicians and high quality patient care [8].

Clinical supervision has been defined as “the formal provision, by approved supervisors, of a relationship-based education and training that is work-focused and which manages, supports, develops and evaluates the work of colleague/s” ( [9] p. 440). Clinical supervision supports effective clinical governance and the professional wellbeing of allied health clinicians [8]. In conjunction with other forms of professional support, clinical supervision improves allied health recruitment and retention, particularly in non-metropolitan settings [10, 11]. Clinical supervision is widely used by allied health professionals. However, there are a number of barriers for effective clinical supervision. These include difficulties accessing clinical supervisors, inconsistent approaches to clinical supervision across different professions and settings and variable effectiveness of clinical supervision across allied health professions [12–15]. Other challenges include poor translation of best practice clinical supervision approaches, such as provision for supervisees to choose their clinical supervisor, and inadequate training of clinical supervisors [16, 17].

Clinical supervision frameworks have been recommended as a policy strategy to improve the effectiveness of clinical supervision for allied health [18–22]. Allied health clinical supervision frameworks can facilitate a common understanding of clinical supervision, agreed roles and responsibilities, and promote consistent approaches [18]. Structured frameworks could also assist health care organisations to evaluate the implementation of clinical supervision and identify areas for improvement [18]. Multidisciplinary allied health clinical supervision frameworks within health service organisations can influence the quality of clinical supervision by promoting the formation of safe and effective supervisory relationships that focus on the professional development of the supervisee [22]. However, internationally, few allied health professions and jurisdictions have developed clinical supervision frameworks and most have been reported to be of low quality [23]. Additionally, there is limited longitudinal research measuring the impact of implemented clinical supervision frameworks [8].

In 2015, a regional health service in Victoria, Australia introduced an organisational clinical supervision framework for allied health. An action research group, comprising of allied health managers at the health service, was established to oversee the implementation and evaluation of the framework. The action research group informed the cycles of planning, action and reflection as a part of the framework implementation. The framework included a multidisciplinary clinical supervision protocol which outlined expectations for clinical supervision and roles and responsibilities. Interprofessional training was conducted to support the implementation of the framework.

When the framework was implemented in 2015, an initial survey was conducted. The findings revealed that while clinical supervision was perceived to be effective overall, there were significant differences in perceived effectiveness between some disciplines [15]. To measure the longer-term perceived effectiveness of clinical supervision and the clinical supervision framework, a repeat survey was planned and undertaken.

The aim of this study was to determine the effectiveness of clinical supervision for allied health at an Australian regional health service and to contrast the findings with those of an earlier survey. A secondary aim was to explore clinicians’ perceptions of the organisational clinical supervision framework.

## Methods

### Research design

A cross-sectional study was conducted as a phase of an overarching participatory action research study. The findings of the survey reported in this paper were compared with the findings of an initial administration of the MCSS-26 survey at the health service 5 years prior [15]. Additional questions were included in the later survey to explore the impact of the organisation’s introduction of the clinical supervision framework and to identify opportunities to enhance clinical supervision. The action research group participated in repeated cycles of planning, action and reflection to inform changes and improvements to clinical supervision at the health service [24]. Four action research cycles were undertaken over a five year period to inform the implementation of the clinical supervision framework, with this study being the focus of the final action research cycle. Consistent with the action research design, the action research group assisted with the interpretation of findings [24]. Ethics approval was provided by Bendigo Health and La Trobe University Ethics Committees (Approval number LNR/15/BHCG/26). All methods were carried out in accordance with the relevant guidelines and regulations and all participants provided informed consent.

### Participants

Participants were allied health professionals and allied health assistants employed at a large regional Australian health service. The allied health professions included were physiotherapy, occupational therapy, social work, dietetics, speech pathology, podiatry, exercise physiology, psychology, audiology and allied health assistants. Participants included a range of staff experience levels and were employed across hospital, community and mental health settings. The survey was distributed electronically by the Director of Allied Health in July 2020 and was open for 5 weeks. To be eligible, participants had to be receiving clinical supervision at the health service for at least 12 months. Participation in the survey was voluntary and data were non-identifiable. The professions were aggregated if they had less than ten responses to reduce the risk of participant identification.

### Outcome measures

To measure perceived effectiveness of clinical supervision, the MCSS-26 tool was used [25]. The MCSS-26 has been validated for use by allied health professionals, has been found to have high test-retest reliability and was the same tool used in the 2015 study [25]. The MCSS-26 is a self-completion tool, measuring the perceived effectiveness of clinical supervision from the supervisee’s perspective [26]. A summary of the MCSS-26 domains and sub-scales is provided in Table 1. The MCSS-26 tool contains 26 questions which are rated on a five point scale from strongly disagree (0) to strongly agree [4]. The possible range of scores is between 0 and 104. Higher scores indicate a greater perceived clinical supervision effectiveness. The developers of the MCSS-26 have recommended that a threshold score of ≥ 73 signifies effective clinical supervision [27]. The MCSS-26 also contains questions relating to the supervisee, their clinical supervision and the clinical supervision session characteristics. Additional closed and open-ended survey questions were included to explore factors influencing the effectiveness of clinical supervision, the impact of the implementation of the health service’s clinical supervision framework and suggestions for improving clinical supervision practice.

**Table 1:** Description of MCSS-26 domains and sub-scales [26]

Normative domain	
Importance/value of clinical supervision: the importance of receiving clinical supervision to improve patient care	
Finding time: the time available for supervisees to participate in clinical supervision	
Formative domain	
Trust/rapport: trust with the supervisor and the ability to raise sensitive or confidential issues	
Supervisor advice/support: the level of support and guidance provided by the clinical supervisor	
Restorative domain	
Improved care/skills: the extent to which clinical supervision improves skills and quality of care	
Reflection: support for the supervisee to reflect on complex issues	

### Data analysis

All available survey data were analysed. Frequency data for valid responses were reported as raw numbers and percentages. MCSS-26 data (total scores, summed domain and sub-scale scores) were analysed descriptively and reported as mean and standard deviation values. Differences between groups were analysed with independent-samples t-test (t) and one-way between groups ANOVA. Due to small numbers, some profession categories were collapsed for comparison of groups. Sub-group analysis included variables of interest informed by the action research group, including supervisor grade level, supervisor allocation, clinical supervision frequency and use of clinical supervision agreements. Total MCSS-26 scores for allied health and each profession in this study were compared descriptively with data from the 2015 study. All quantitative analyses were performed using SPSS version 26. Open ended survey question data were thematically coded using content analysis by two researchers independently and then discussed together to reach consensus [28].

## Results

### Responses

One hundred and twenty-five allied health clinicians responded to the survey, with a response rate of 50.0% of eligible participants.

### Participant characteristics

The responding participants were predominantly female (*n* = 102; 81.6 %) and the mean age was 38.2 years (S.D. 11.5). Over half of the participants were in grade 2 positions (*n* = 67; 53.6%), early to mid-career clinicians with generally 2 to 7 years of experience. Nearly a quarter were at grade 3 or 4 levels (*n* = 30; 24.4%), mainly senior clinicians with more than 7 years of experience. Approximately one sixth were in grade 1 positions (*n* = 19; 15.2%), graduates and early career clinicians generally with up to 2 years’ experience. There was a similar number of participants working in hospital (*n* = 48; 38.4 %) and community settings (*n* = 51; 40.8 %), with the remainder working across both. Approximately half of the participants had attended clinical supervision training (*n* = 61; 48.8 %). Participant characteristics are summarised in Table 2.

**Table 2:** Supervisee characteristics (*n* = 125)

	No. of participants	%
**Sex**		
Male	18	14.4
Female	102	81.6
Missing	5	4.0
**Staff Grade**		
Grade 1	19	15.2
Grade 2	67	53.6
Grade 3 and 4	30	24.0
Management	3	2.4
Other	1	0.8
Missing	5	4.0
**Discipline**		
Occupational therapy	36	28.8
Physiotherapy	33	26.4
Social work	22	17.6
Dietetics	7	5.6
Speech pathology	10	8.0
Psychology	2	1.6
Podiatry	3	2.4
Exercise physiology	4	3.2
Other	1	0.7
allied health Assistants	2	1.5
Missing	5	4.0
**Work setting**		
Hospital	48	38.4
Community	51	40.8
Both	21	16.8
Missing	5	4.0
**Supervisor allocated or chosen**		
Allocated	104	83.2
Chosen	12	9.6
Other	3	2.4
Missing	6	4.8
**Attended training**		
Yes	61	48.8
No	56	44.8
Missing	8	6.4

### Clinical supervision session characteristics

Over half of the participants’ clinical supervisors were senior clinicians (grade 3 or 4) (*n* = 64; 52.5%) while approximately a quarter were in management (*n* = 29; 23.8%) or grade 2 positions (*n* = 26; 21.3%). Most participants had clinical supervisors who were allocated to them (*n* = 104; 83.2 %). Clinical supervision sessions mostly occurred monthly, within the workplace and for under one hour. Most participants received individual clinical supervision (*n* = 115; 83.3 %), with a smaller number participating in group clinical supervision (*n* = 14; 10.7%) and 75% had a clinical supervision agreement in place (*n* = 94). Characteristics of supervision sessions are provided in Table 3.

**Table 3.** Characteristics of supervision sessions (*n* = 125)

	No. of participants	%
**Frequency of supervision sessions**		
Weekly	1	0.8
Fortnightly	12	9.6
Monthly	82	65.6
2-3 months	18	14.4
>3 months	6	4.8
Missing	6	4.8
**Location of supervision session**		
Within the workplace	99	79.2
Away from the workplace	6	4.8
Both	14	11.2
Missing	6	4.8
**Type of supervision**		
One to one	115	83.3
Triad	3	2.2
Group	14	10.7
Other	6	4.3
**Duration of supervision sessions**		
<15 mins	1	0.8
15-30 mins	8	6.4
31-45 mins	12	8.0
46-60 mins	84	67.2
>60 mins	18	12.0
Missing	2	1.6
**Clinical supervision agreement in place**		
Yes	94	75.2
No	23	18.4
Missing	8	6.4

### MCSS-26 results

Clinical supervision was perceived by participants to be effective overall (Table 4). The average total MCSS-26 26 score across all professions was 78.5 (S.D. 14.5). Scores in the *formative* domain were highest of the three domains, with the sub-scale relating to *reflection* scoring highest. The lowest domain score was for the *normative* domain. Of the two sub-scales in the *normative* domain, there was a relatively high score for the *importance/value* sub-scale and a lower score for *finding time* sub-scale.

**Table 4:** MCSS-26 total, domain and sub-scale scores for all allied health professions

MCSS-26 scores	No. of items	Possible range	Mean (S.D.)	Mean (out of 100) (+/- S.D.)^**a**^
**Normative domain**	9	0-36	26.1 (5.4)	72.5 (5.2)
Importance/value of clinical supervision	5	0-20	16.3 (2.9)	81.5 (2.8)
Finding time	4	0-16	9.8 (3.3)	61.2 (3.2)
**Formative domain**	7	0-28	21.8 (4.5)	77.9 (4.3)
Improved care/skills	4	0-16	12.2 (2.9)	76.2 (2.8)
Reflection	3	0-12	9.6 (2.1)	80.0 (2.0)
**Restorative domain**	10	0-40	30.6 (6.9)	76.5 (6.6)
Trust/support	5	0-20	15.7 (3.2)	78.5 (3.1)
Supervisor support/advice	5	0-20	14.8 (4.2)	74.0 (4.0)
**Total**	26	0-104	78.5 (14.5)	75.5 (13.9)

^a^Mean expressed out of 100 to enable comparison across domains and subscales

Participants whose clinical supervisor was at a grade 3 or 4 level (82.0; S.D. 12.4) had higher MCSS-26 scores than those whose clinical supervisor was a manager (73.7; S.D. 17.2, F = 2.8, p = 0.04). There were higher MCSS-26 scores for those who had input into choosing their clinical supervisors (86.6; S.D. 11.58) than those who did not (78.2; S.D. 13.8, t = -2.3, p = 0.047). Those who had clinical supervision monthly or more frequently (80.7; S.D. 14.2) had higher scores than those who had clinical supervision every 2 months or more (71.5; S.D. 9.7, F = 4.7, p = 0.01). Attendance at training and use of clinical supervision agreements did not influence MCSS-26 scores.

Scores were higher for speech pathologists (88.5; S.D. 10.2), social workers (81.1; S.D. 16.7) and occupational therapists (80.4, S.D. 10.2) compared to physiotherapists (74.9; S.D. 8.9) and clinicians in the “other” group of professions (73.9; S.D. 13.9). ANOVA analysis for the impact of the supervisee’s profession on MCSS-26 scores demonstrated a statistically significant difference between speech pathology and physiotherapy (F = 2.9, p = 0.03). There were no other significant differences between professional groups.

### Impact of organisational clinical supervision framework

Seventy percent of participants agreed or strongly agreed that the organisation’s clinical supervision framework had been useful. Twenty-two percent were neutral while 8% disagreed. Themes from open-ended responses relating to the impact of the framework included the framework *provides structure and consistent expectations*, *lack of awareness* of the framework, and the need for *framework modifications*. Some participants indicated that the framework *provides structure and consistent expectations* for clinical supervision practice*,* while in contrast others had a *lack of awareness* of the framework or perceived that it had made no change. Some responses indicated the need for *framework modifications*, such as redesigning clinical supervision documentation templates.

Considering future improvements to the organisational clinical supervision framework, themes associated with responses included *no changes*, *choice of clinical supervisor*, *group or peer models*, *access to clinical supervision education and training*, and *clarity around the purpose of clinical supervision.* The themes arising from those who proposed improvements were: having input into the *choice of clinical supervisor*; increased opportunities for *group or peer models* of clinical supervision; increased *access to clinical supervision education and training*; and improved *clarity around the purpose of clinical supervision*, particularly for senior clinicians and those in specialist roles.

### Comparison of 2020 results with 2015 results

The total MCSS-26 mean score for all allied health professions did not change between 2015 (78.5, S.D. 13.9) and 2020 (78.5, S.D. 14.5) results (see Table 5). The restorative domain score increased from 2015 (30.0, S.D. 7.3) to 2020 (30.6, S.D. 6.9) and the normative domain score decreased from 2015 (26.6, S.D. 4.7) to 2020 (26.1, S.D. 5.4). The formative domain mean score was unchanged. The total MCSS-26 score for physiotherapy increased from 2015 (70.9, S.D. 11.3) to 2020 (74.9, S.D. 8.9).

**Table 5:** Comparison of MCSS-26 total, domain and sub-scale scores for all disciplines, 2015 and 2020

	2015 survey, mean (SD)	2020 survey, mean (SD)
**Normative domain**	**26.6 (4.7)**	**26.1 (5.4)**
Importance/value of clinical supervision	16.9 (2.4)	1*6.3 (2.9)*
Finding time	9.7 (3.2)	9.8 (3.3)
**Formative domain**	**21.8 (4.6)**	**21.8 (4.5)**
Improved care/skills	12.4 (2.8)	12.2 (2.9)
Reflection	9.4 (2.3)	9.6 (2.1)
**Restorative domain**	**30.0 (7.3)**	**30.6 (6.9)**
Trust/support	15.1 (3.6)	15.7 (3.2)
Supervisor support/advice	14.9 (4.3)	14.8 (4.2)
**Total**	**78.5 (13.9)**	**78.5 (14.5)**

Remaining professional group scores were similar between 2015 and 2020, apart from the “other profession” group which decreased from 2015 (83.6, S.D. 12.2) to 2020 (73.9, S.D. 13.9), however, there were different professions included in the “other profession” group in 2015 and 2020. Table 6 provides a comparison MCSS-26 scores for discipline groups between the two administrations of the survey.

**Table 6:** Total MCSS-26 scores for individual allied health professions, 2015 and 2020

Discipline	Mean (SD), 2015	Mean (SD), 2020
**Social work**	81.3 (13.6)	81.1 (16.7)
**Physiotherapy**	70.9 (11.3)	74.9 (8.9)
**Occupational therapy**	82.8 (14.4)	80.4 (10.2)
**Speech pathology**^**a**^	*-*	88.5 (10.2)
**Dietetics**^**b**^	70.4 (12.0)	*-*
**Other professions**^**c**^	83.6 (12.2)	73.9 (13.9)
**Allied health**	78.5 (13.9)	78.5 (14.5)

^a^insufficient number of responses in 2015 to analyse speech pathology separately (included in “other professions” group in 2015)

^b^insufficient number of responses in 2020 to analyse dietetics separately (included in “other professions” group in 2020)

^c^other profession group in 2015 included: speech pathology, psychology, podiatry, exercise physiology and allied health assistance; other profession group in 2020 included: dietetics, psychology, podiatry, exercise physiology and allied health assistance

## Discussion

Clinical supervision was perceived by supervisees to be effective overall. All professions with more than ten responses had MCSS-26 scores above the threshold score for effective clinical supervision of 73. There was variable effectiveness of clinical supervision across allied health professions. The overall results were similar to those reported in other allied health studies using the MCSS-26, however, the score for speech pathology was higher than in previous studies [14, 29, 30]. This novel finding contrasts with previous studies. In other studies the professions with the most effective clinical supervision were counselling-based professions with more established traditions of clinical supervision, such as social work and psychology [14, 29]. The lower score for physiotherapy is consistent with several other studies [14, 18, 19]. This may be explained by other research findings where physiotherapists reported greater satisfaction when direct models of clinical supervision are used rather than a mix of direct and reflective clinical supervision [31, 32] which was the predominant model used by physiotherapists in this study.

The effectiveness of clinical supervision in this study was influenced by the level of experience and seniority of the clinical supervisor. Clinical supervision was most effective when provided by a senior clinician (grade 3) and least effective when provided by a manager. This finding builds on existing evidence that clinical supervision should be separated from line management to prevent blurring the boundaries of these functions and clinical supervision becoming overly focused on administrative functions [16, 22, 33–35]. Additionally, there is a power differential when a manager is the clinical supervisor which may result in “supervisee guarding” where supervisees avoid discussing issues around work skills and performance for fear of being viewed as incompetent [36].

Managers should also participate in clinical supervision that is separated from operational accountability. This provides opportunities to maintain and develop their own clinical supervision skills and increase their access to professional support. Strategies suggested by participants in this study to address this issue included implementing peer group models of clinical supervision for senior clinicians which could ensure that clinical supervision is separated from operational issues. Another potential strategy was cross-organisational models of clinical supervision for senior clinicians and clinical managers. This could enable experienced clinicians to access clinical supervision that is relevant to their clinical speciality or role and promote sharing of evidence-based practice between organisations. Facilitating cross-organisational approaches would require the development of coordinated jurisdictional clinical supervision policies to address issues such as establishing registers of clinical supervisors and cost-sharing between organisations.

In this study, another factor associated with increased effectiveness of clinical supervision was when supervisees chose their clinical supervisor. The theme of clinicians wanting to have input into choosing their supervisor was present in responses from participants suggesting improvements for clinical supervision. This finding is consistent with the recommendations made by other researchers as best practice clinical supervision [37, 38]. Aside from social workers who had a process for enabling supervisees to choose supervisors, the health service’s model of supervision was based on hierarchical allocation of supervisors within the same profession and speciality. This is a similar model of supervisor allocation reported within health services in other allied health clinical supervision studies [14, 29]. When discussing this finding, the action research group determined that it was not practical to enable all staff to choose their supervisor within the existing allied health structures in this health service. This is supported by a study involving allied health in community health settings that reported that workforce structures containing insufficient numbers of senior clinicians was a barrier to accessing and allocating clinical supervisors [12]. However, it may be possible for some clinicians, such as experienced clinicians or those who have had the same supervisor for an extended period, to have input into the choice of their supervisor.

The overall MCSS-26 score in this study for the health service was the same as the score reported in the 2015 study, indicating that the overall effectiveness of clinical supervision was maintained during the 5 year period between surveys. There were minimal changes in the domain and sub-scale scores with the sub-scales relating to the importance of clinical supervision and its role in assisting reflection scoring higher in both surveys and finding time for clinical supervision scoring the lowest in both. The lack of change in overall effectiveness of clinical supervision could reflect that the introduction of the framework made no difference. Another interpretation could be that, due to the increased focus on clinical supervision, clinicians had a greater awareness of its benefits and therefore had higher expectations for the effectiveness of clinical supervision provided to them.

There were increases in scores between surveys for some professions. While the 2015 study did not include enough responses from speech pathology to enable this profession to be analysed individually, the high score for speech pathology in 2020 was notable. The action research group reflected that the high score for speech pathology may be associated with improved clarity around the purpose of clinical supervision as well as an overall emphasis on developing a culture of learning in this profession. There was also a modest increase in the score for physiotherapy. Neither speech pathology nor physiotherapy had structured frameworks for clinical supervision in place prior to the implementation of the organisational framework in 2015, which may have contributed to the increased effectiveness of clinical supervision for these professions. In contrast, social work and occupational therapy, who did have existing frameworks in place prior to the introduction of the organisational framework, had no change or a slight decrease in their MCSS-26 scores.

Participants perceived that the organisational framework provided structure and clear expectations for clinical supervision. Previous studies have recommended that structured frameworks for clinical supervision are required to improve the quality of clinical supervision [18, 19]. However, this study is the first that the authors are aware of to provide a longitudinal comparison of the impact of implementing a clinical supervision framework for allied health, demonstrating that such initiatives can have a positive impact on clinical supervision. This finding strengthens the need for future policy direction to focus on the development of common allied health clinical supervision frameworks to facilitate consistent approaches to clinical supervision implementation and education.

Whilst the response rate in this study was 50%, it was within the range of response rates reported in other studies using the MCSS-26 survey (167 29, 30). However, the low number of responses in some professions restricted the analysis of comparison between groups and there may have been a positivity bias amongst those who responded. Additionally, the ability to draw conclusions when comparing the 2015 and 2020 survey findings was limited as a formal pre-post design was not used, consistent with recommendations for the use of MCSS-26 and an action research approach.

## Conclusion

Clinical supervision was effective for allied health in this regional setting and effectiveness been maintained over a 5 year period. Strategies to improve clinical supervision practice in the future include providing opportunities for supervisees to choose their clinical supervisor and introducing peer group supervision for experienced clinicians. The implementation of an organisational clinical supervision framework may have a positive effect on clinical supervision for some professions. There is a need for future policies to focus on the development of common, structured frameworks to support quality clinical supervision across allied health.

## Data Availability

The datasets generated and analysed during the current study are not publicly available due to conditions of ethical approval. However, a de-identified version of the data is available from the corresponding author on reasonable request.
